# Supplementary material for: Genetic regulatory effects modified by immune activation contribute to autoimmune disease associations
Source: Nat Commun. 2017 Aug 16;8:266. doi: 10.1038/s41467-017-00366-1 (PMC5559603; doi:10.1038/s41467-017-00366-1)
Supplement: Supplementary file 1 — Supplementary Information [file 41467_2017_366_MOESM1_ESM.pdf]

File Name: Peer Review File

File Name: Supplementary Information
Description: Supplementary Figures, Supplementary References.

File Name: Supplementary Data 1
Description: List of differential expression and enriched pathways after stimulation of primary human monocytes.

File Name: Supplementary Data 2
Description: List of genome-wide significant eQTLs (FDR 5%).

File Name: Supplementary Data 3
Description: Coloc results and list of GWAS traits used in this study.

File Name: Supplementary Data 4
Description: List of GWAS reQTLs.

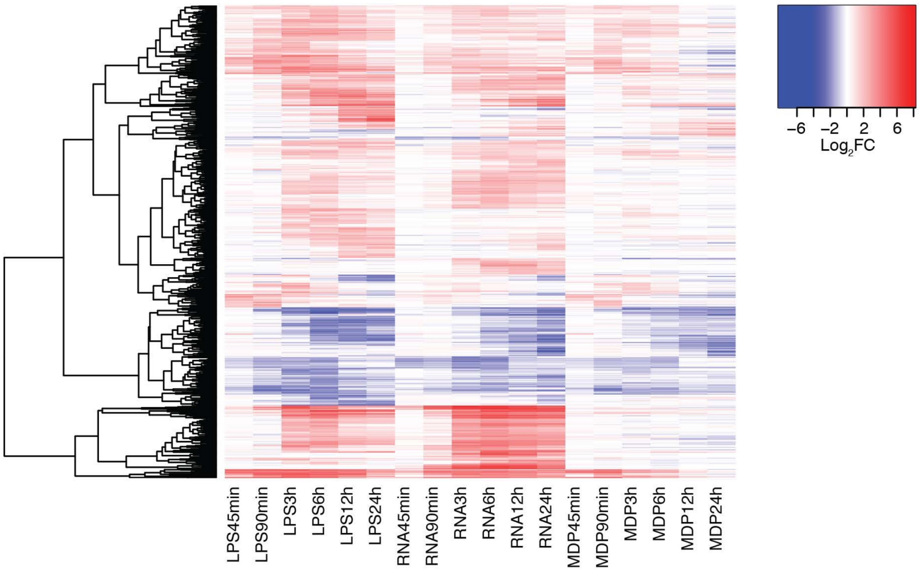

Supplementary Figure 1. Pilot time course experiment in stimulated monocytes of five individuals. Heatmap of differentially expressed genes (log_2-fold change > 1, FDR 0.001) from monocytes stimulated with LPS, ppp-dsRNA or MDP over a detailed time course of 45min, 90min, 3h, 6h, 12h and 24h. Genes are hierarchically clustered. Time points where early response genes (90 min) and late response genes (6 h) showed robust differential expression were used for subsequent experiments.

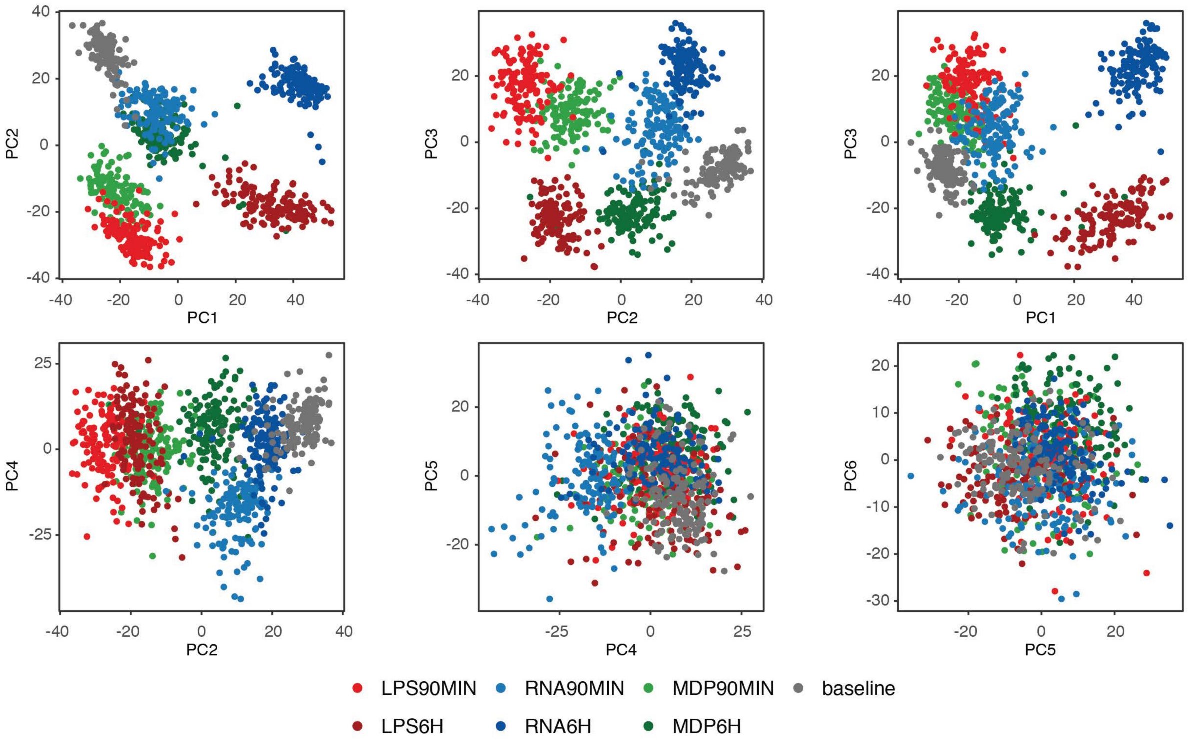

Supplementary Figure 2. Principal components analysis. Principal components analysis of expression data from 134 individuals used in the eQTL analysis. The first 3 principal components (PC) account for inter-condition effects, with no clustering along conditions observed in subsequent PCs.

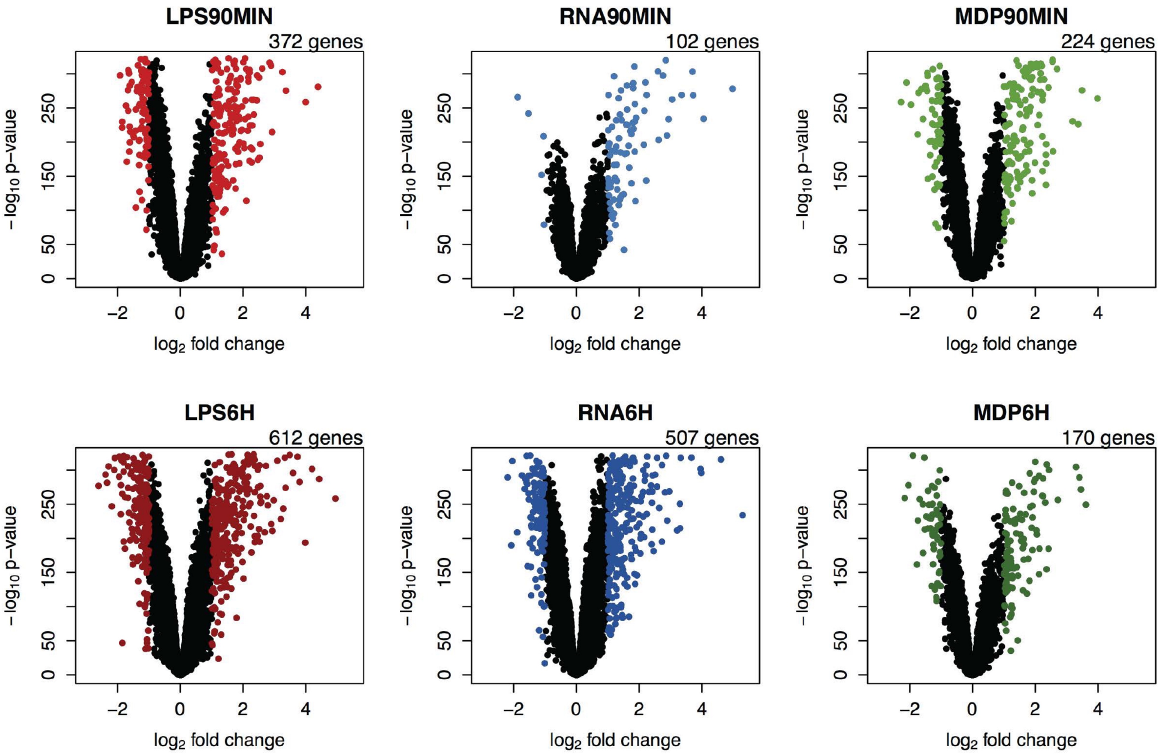

Supplementary Figure 3. Differential expression in stimulated monocytes. Volcano plots showing differentially expressed transcripts upon immune stimulation. The negative $\log_{10}$ transformed *p* values test the null hypothesis of no difference in expression levels between stimulated and unstimulated monocytes (y axis) and are plotted against the average $\log_2$ fold changes in expression (x axis). Genes that are differentially expressed (DE) upon stimulation with a mean $\log_2$ fold change > 1 are color-coded with the treatment, and the number of DE genes is indicated at the top of each plot.

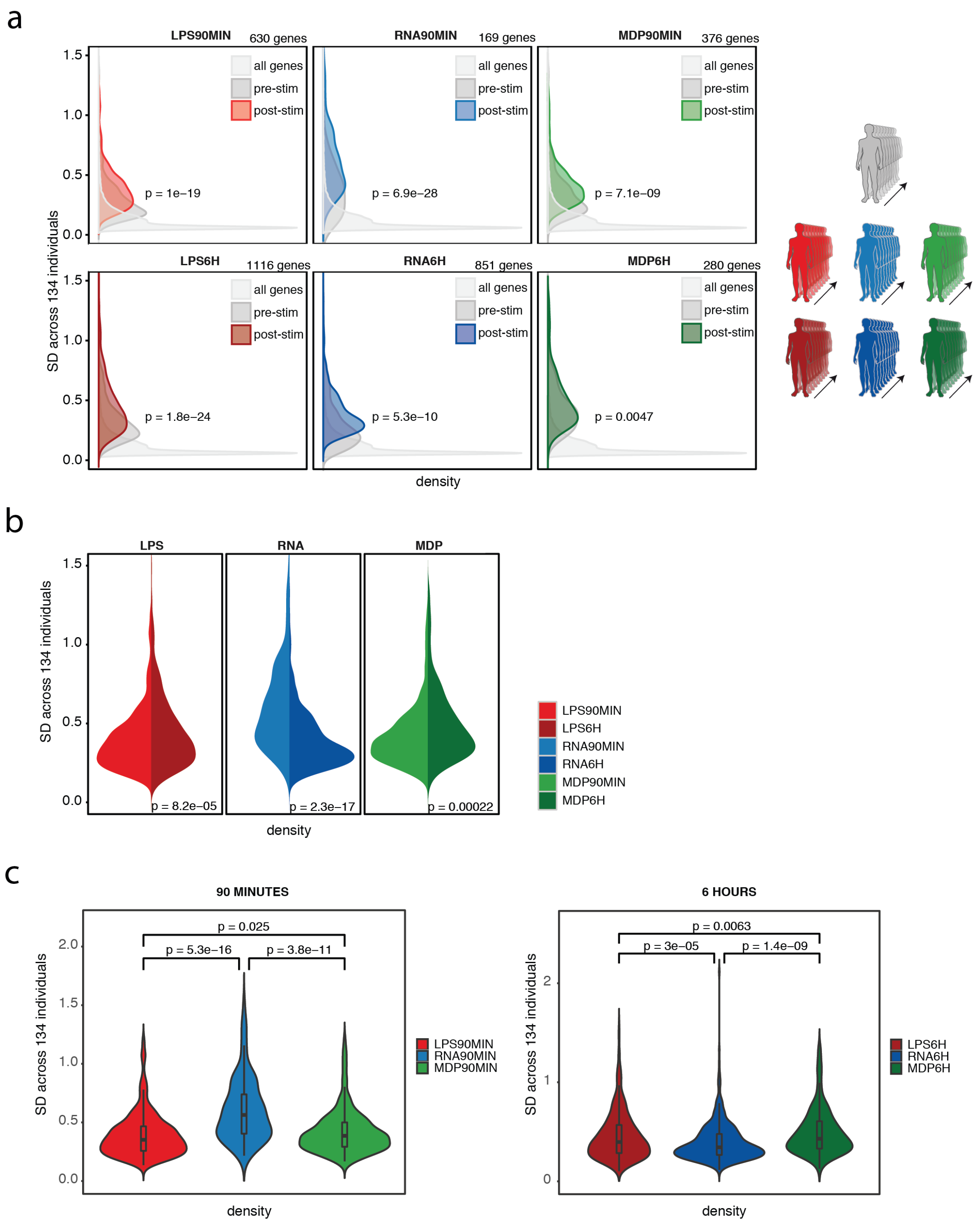

Supplementary Figure 4. Interindividual variability of immune response genes across 134 individuals. (a) Density plot of standard deviation (SD) of DE genes from Supplementary Fig. 3 before and after stimulation. Distributions of SD of all genes are shown in light grey. Difference in the distribution of SD before and after stimulation were tested using Wilcoxon rank-sum test. **(b)** Violin plots of SD after stimulation comparing two time points of each stimulus or **(c)** all three treatments of each time point. Differences in SD in each plot were tested using Wilcoxon rank-sum test.

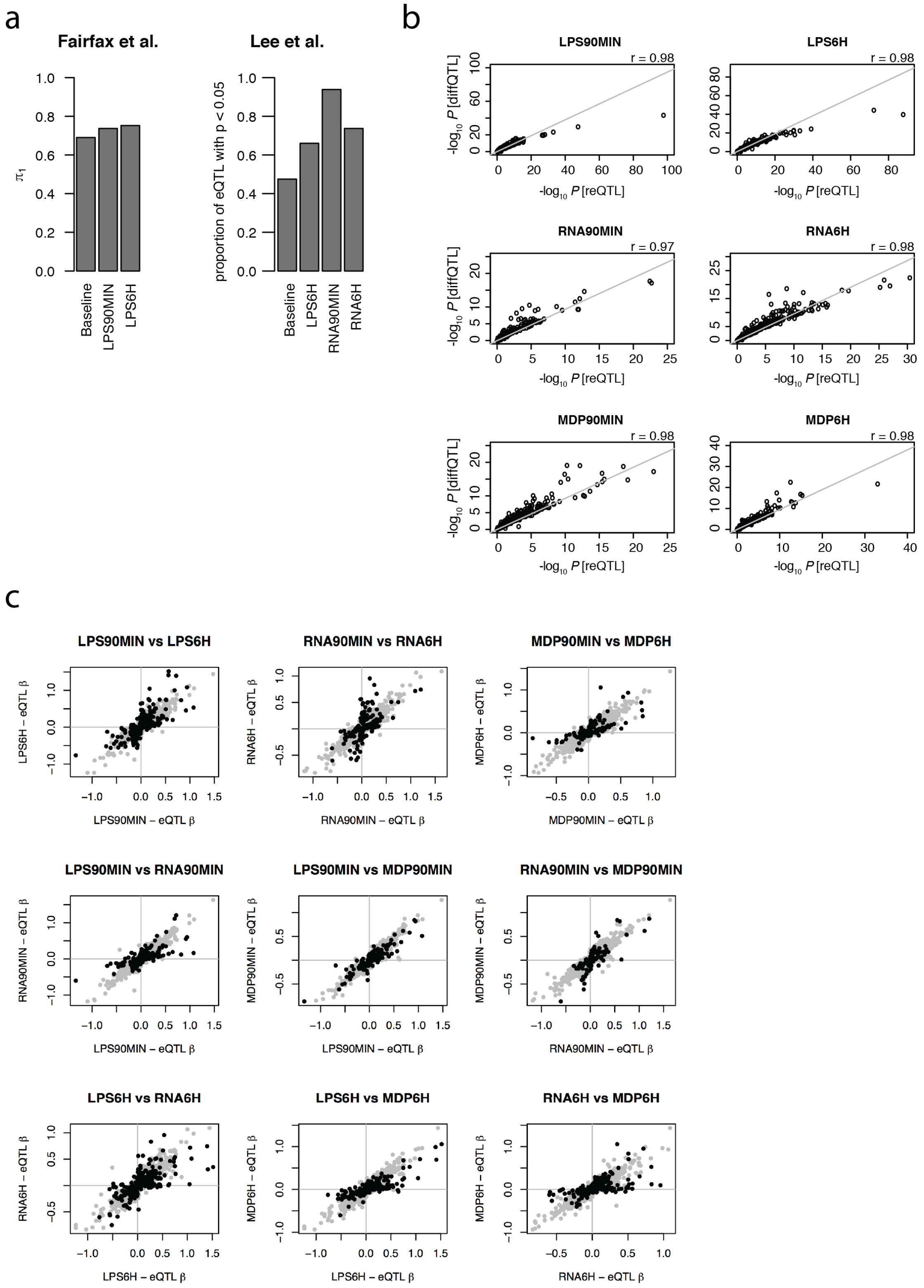

Supplementary Figure 5. Robustness of eQTLs identified in this study. (a) The study dataset was compared with two publically available eQTL datasets under baseline and under immune stimulation. For the left panel we used Fairfax et al.[1], whole transcriptome expression microarray data in human primary monocytes unstimulated, or stimulated with LPS for 2 hours or 24 hours or the cytokine IFNγ for 24 hours. Our baseline eQTLs were compared with eQTLs from unstimulated monocytes of Fairfax et al., our LPS90min eQTLs with Fairfax's LPS 2 hours, and our

LPS6h eQTLs with Fairfax's LPS 24 hours. Bar chart shows π_1 to quantify eQTL replication. Right panel: Bar chart showing percentage of eQTLs from our dataset with nominally significant p-values in Lee et al. [2] that had eQTLs from 415 genes in human primary dendritic cells (DCs) unstimulated, or stimulated with LPS, influenza virus, or the cytokine IFN-β. Due to the low number of overlapping eGenes and the gene set, π_1 could not be calculated. To match conditions, we compared our baseline eQTLs with eQTLs from unstimulated DCs, our LPS6h eQTLs with Lee's LPS 5 hours, and our RNA90min and RNA6h using Lee's Influenza 10 hours and IFN-β 6.5 hours since both pathways are induced by our RNA stimulation. The mean proportion of the comparison with Influenza 10 hours and IFN-β 6.5 hours is shown in the bar chart. **(b)** Scatter plot of immune response eQTL p-values calculated using beta comparison (reQTL) on the x-axis versus differential expression as phenotype (diffQTL) on the y-axis. Spearman's rank correlation coefficient is shown in the upper right corner of each plot. **(c)** Comparison of eQTL β directions between different treatment conditions. All significant eQTLs (FDR 5%) are shown in grey. eQTLs that are reQTLs in any of the two plotted conditions are highlighted in black.

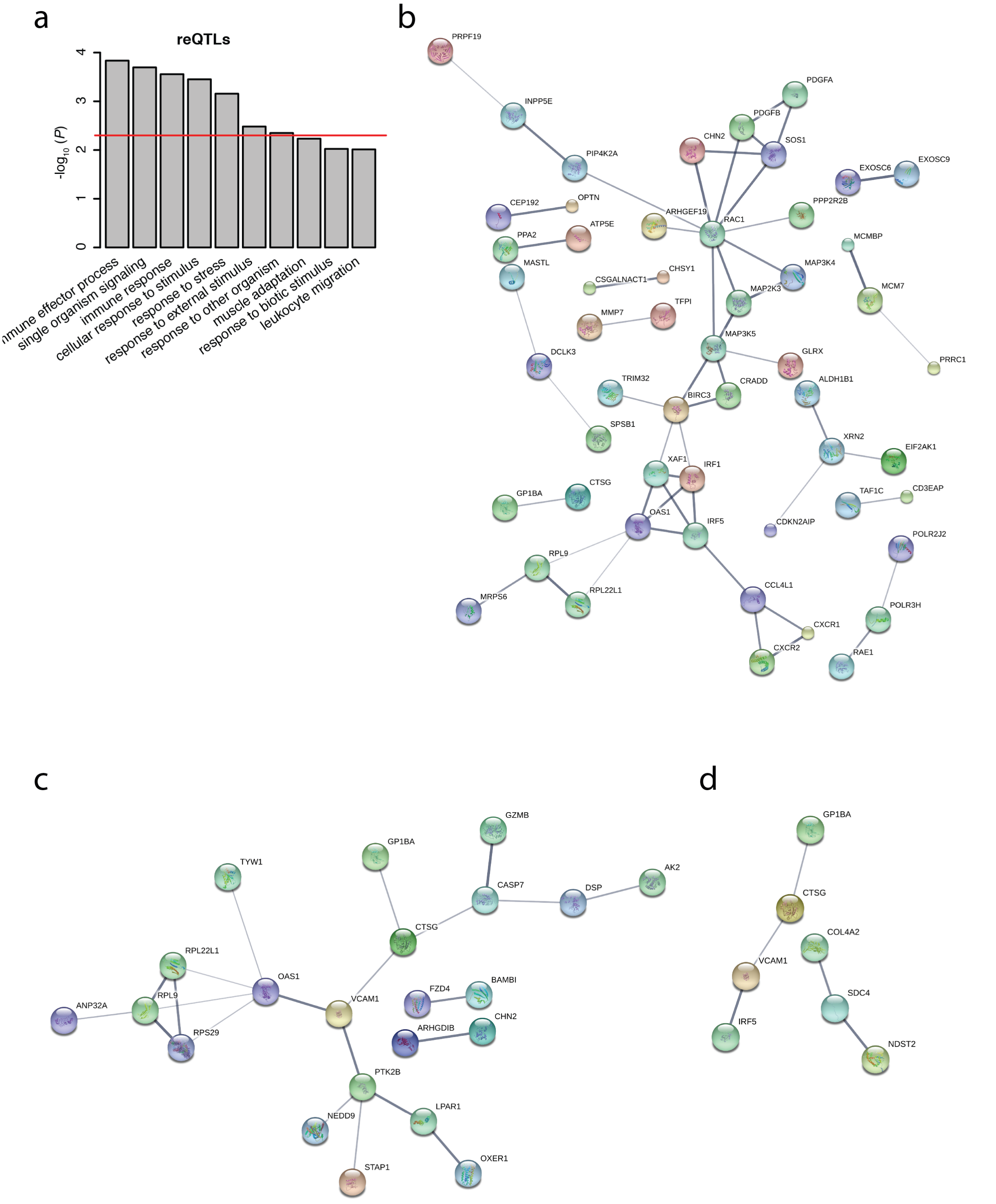

Supplementary Figure 6. reQTL genes are enriched in core networks of the innate immune system. **(a)** Gene ontology enrichment analysis of reQTL genes using ConsensusPathDB[3] and all expressed genes of our monocyte data set as the background. Qvalue 0.05 is indicated by the red line. **(b)** STRING[4]-based PPI network of reQTL genes identified upon LPS90min and LPS6h stimulation. Enlarged nodes indicate the availability of 3D protein structure information. Line thickness between the proteins indicates the strength of data support considering only interactions that were either experimentally validated or originated from curated databases. Disconnected reQTL genes are not shown. **(c)** PPI network of reQTLs

identified upon RNA90min and RNA6h stimulation and **(d)** MDP90min and MDP6h stimulation.

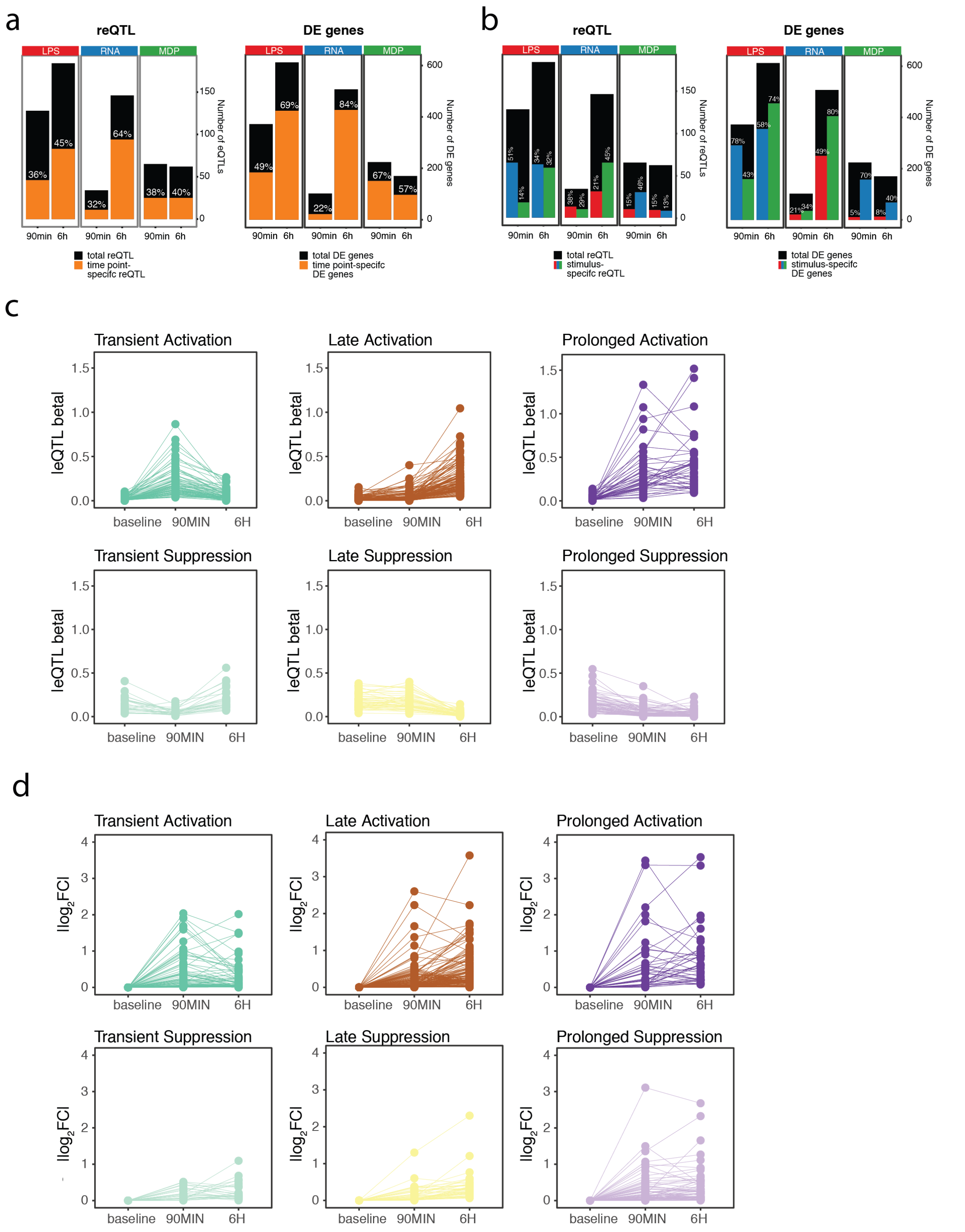

Supplementary Figure 7. Dynamics of immune reQTLs in stimulated monocytes. (a) Proportion of time point specificity of reQTLs versus differentially expressed (DE) genes. Left panel: Bar chart of number of reQTLs and proportion of time point-specific reQTLs. Right panel: Bar chart of number of DE genes and proportion of time point specific DE genes. **(b)** Proportion of treatment specificity of reQTLs versus differentially expressed (DE) genes. Left panel: Bar chart of number of reQTLs and proportion of treatment-specific reQTLs. Right panel: Bar chart of

number of DE genes and proportion of treatment-specific DE genes. **(c)** reQTLs were divided into seven subsets according to their temporal activity. Absolute effect sizes of each eQTL are shown for each category. The average per category is shown in Fig. 2d. **(d)** Corresponding absolute $\log_2$ fold change of gene expression of reQTL genes in the same groups as in **(c)**.

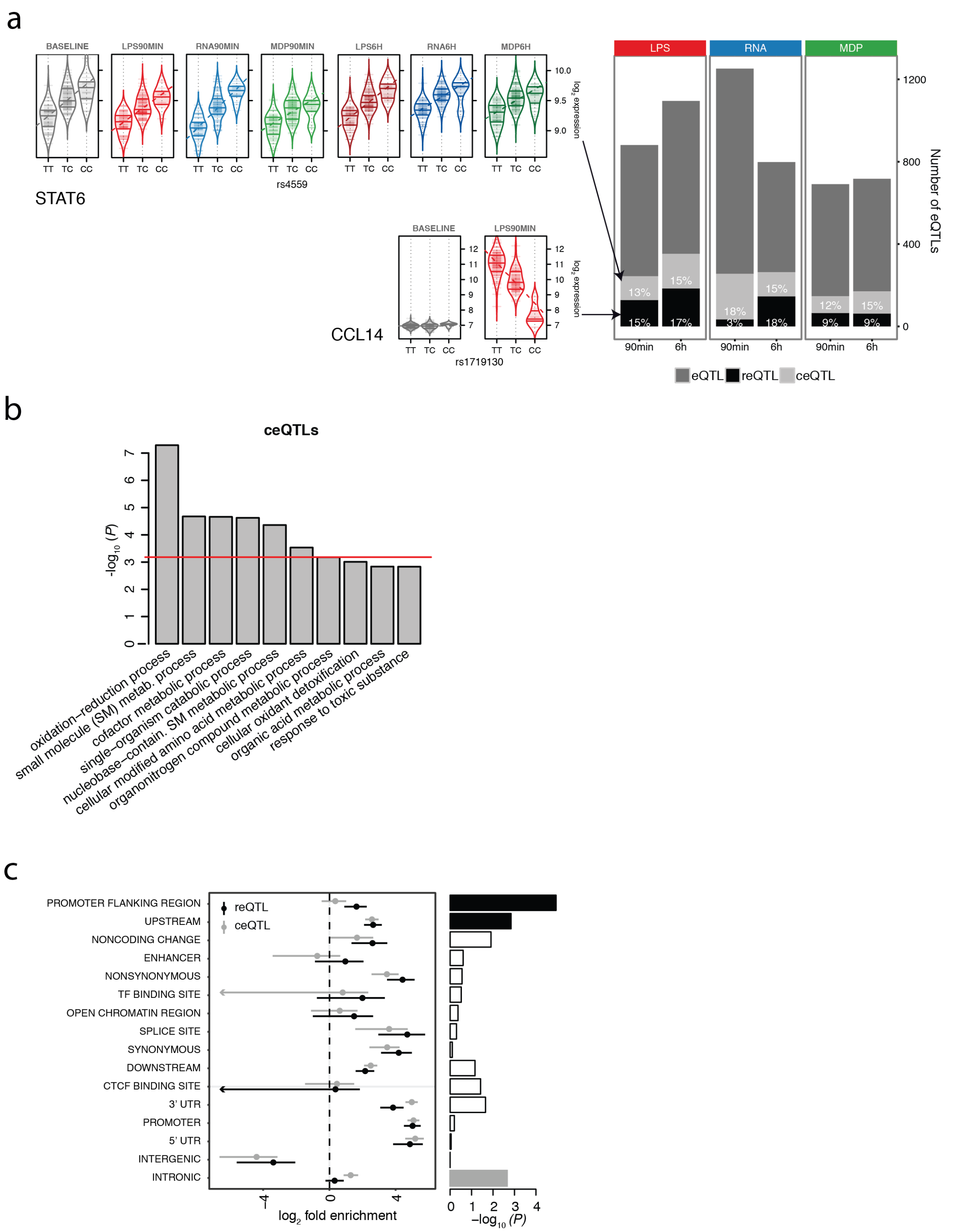

Supplementary Figure 8. Annotations of reQTLs and constant eQTLs (ceQTLs). (a) Same bar chart as in Fig. 3a of the total number of *cis* eQTLs, proportion of ceQTLs and proportion of reQTLs of LPS-treated, ppp-dsRNA-treated and MDP-treated monocytes at 90min and 6h after stimulation. Violin plots for *STAT6* and *CCL14* are shown as examples of ceQTLs and reQTLs, respectively. **(b)** Gene ontology enrichment analysis of ceQTL genes using ConsensusPathDB[3] and all expressed genes of our monocyte data set as the background. Qvalue 0.05 is

indicated by the red line. See Supplementary Fig. 6a for reQTL gene ontology analysis. **(c)** Enrichment of annotations from fgwas using eQTLs mapped by using the mean of all seven conditions as phenotype in eQTL mapping to increase power, followed by classification of the eQTLs to the same reQTL and ceQTL groups as before. Forest plot shows enrichment estimates with 95% confidence intervals for each annotation used in fgwas. x-axis is truncated at -6 for ease of display. Bar plot shows a comparison of reQTL and ceQTL enrichments of the single most likely causal SNP per locus after fine mapping of reQTLs and ceQTLs. Black bars indicate annotations significantly overrepresented in reQTLs after Bonferroni correction. Grey bars indicate annotations significantly overrepresented in ceQTLs after Bonferroni correction.

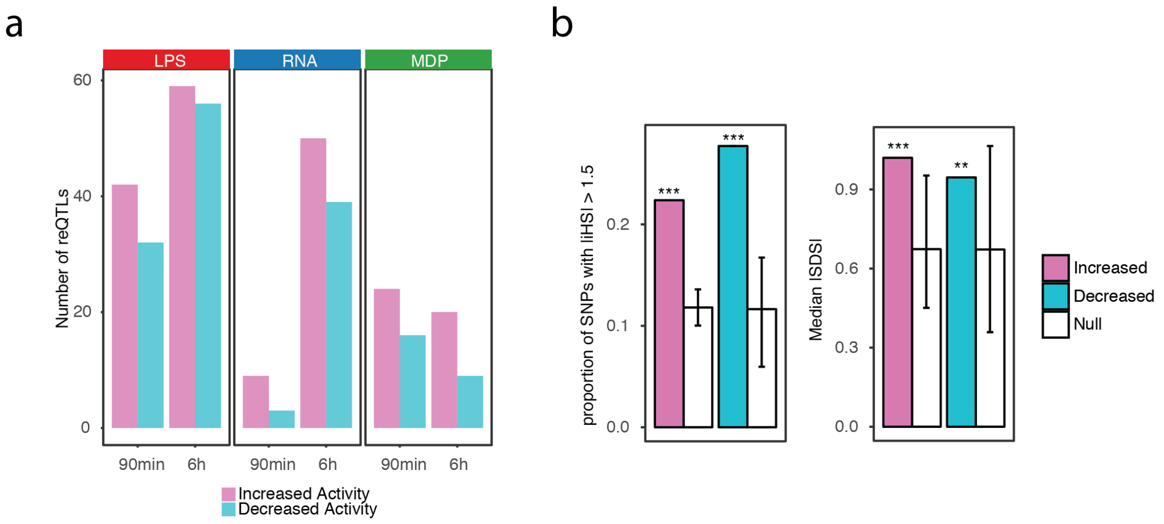

Supplementary Figure 9. Trend of enhanced immune response driven by derived reQTL alleles. (a) Bar chart of the proportion of reQTL with increasing (pink) or decreasing (blue) activity shown for each stimulated condition. The sum of reQTLs with increasing or decreasing activity across all conditions is shown in Fig. 3e. **(b)** For each locus, the maximum |iHS| (left panel) or maximum |SDS| (right panel) across all SNPs in high LD (r^2 > 0.8) is considered respectively. Genome-wide null sets of variants matched to reQTLs with increasing or decreasing activity were generated by resampling 10,000 sets of random SNPs that matched for MAF and LD (white bars). Error bars indicate minimum and maximum of the null distribution. Asterisks represent the significance of enrichment at observed versus randomized matched SNPs (permutation test **p < 0.001; ***p < 10^{-4}).

a

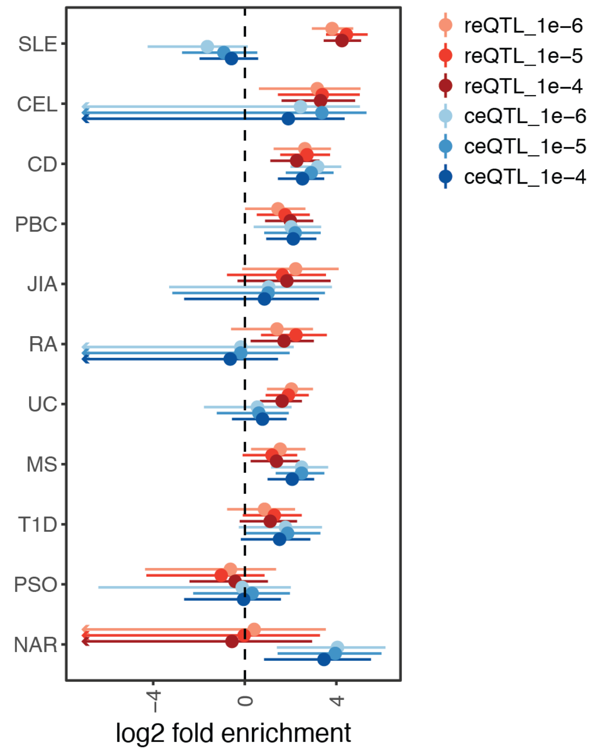
SLE
CEL
CD
PBC
JIA
RA
UC
MS
T1D
PSO
NAR
reQTL_1e−6
reQTL_1e−5
reQTL_1e−4
ceQTL_1e−6
ceQTL_1e−5
ceQTL_1e−4
−4
0
4
log2 fold enrichment

b

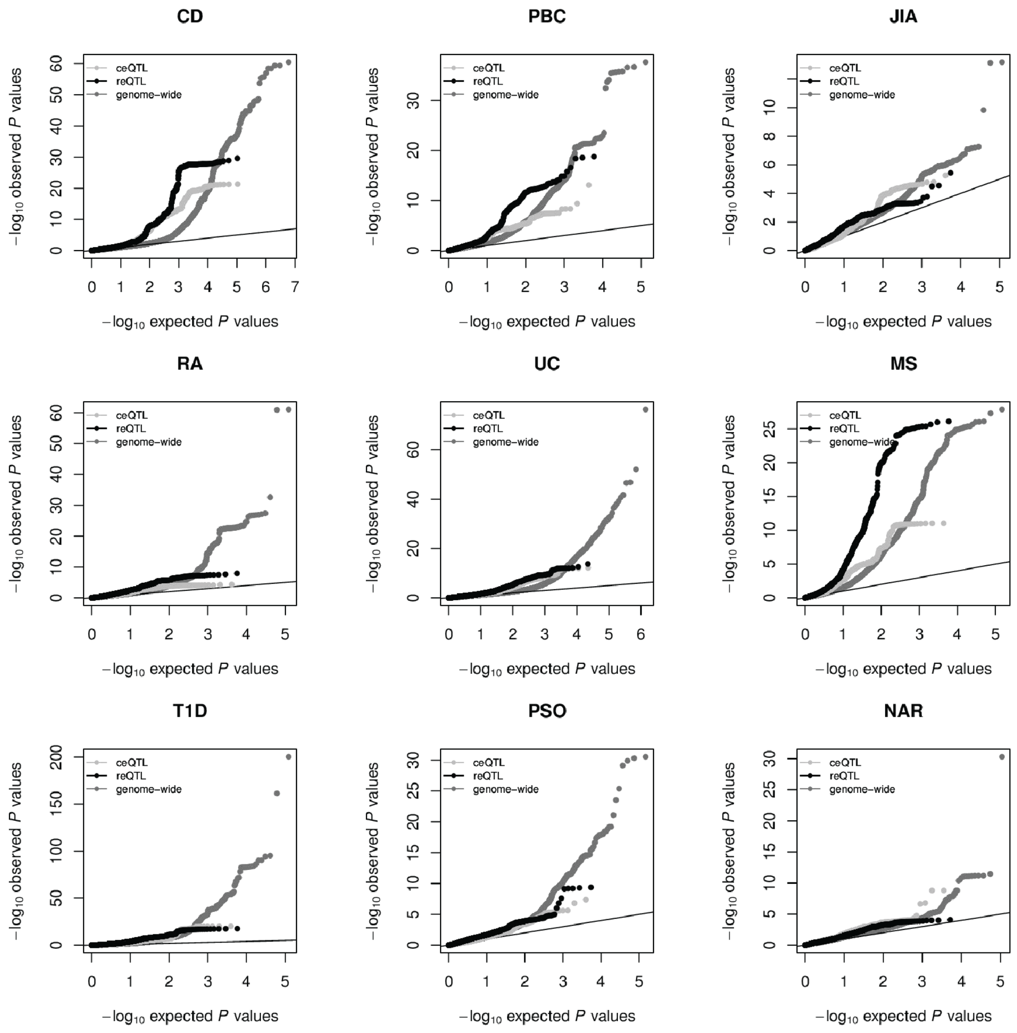
CD
PBC
JIA
RA
UC
MS
T1D
PSO
NAR
ceQTL
reQTL
genome−wide
−log10 observed P values
−log10 expected P values

Supplementary Figure 10. Contribution of reQTLs to autoimmune traits. (a) Genome-wide enrichment analysis of reQTL and ceQTL associations in autoimmune GWAS using various eQTL p-value cutoffs yields similar estimates. x-axis is truncated at -6 for ease of display. Trait abbreviations: Systemic lupus erythematosus (SLE), celiac disease (CEL), crohn's disease (CD), primary biliary cirrhosis (PBC), rheumatoid arthritis (RA), ulcerative colitis (UC), multiple sclerosis (MS), juvenile idiopathic arthritis (JIA), type I diabetes (T1D), psoriasis (PSO) and narcolepsy (NAR). **(b)** Q-Q plots for the remaining 9 autoimmune traits tested for enrichment of reQTLs and ceQTLs. All hits in the extended MHC region (26Mb–34Mb on hg19 chromosome 16) were removed.

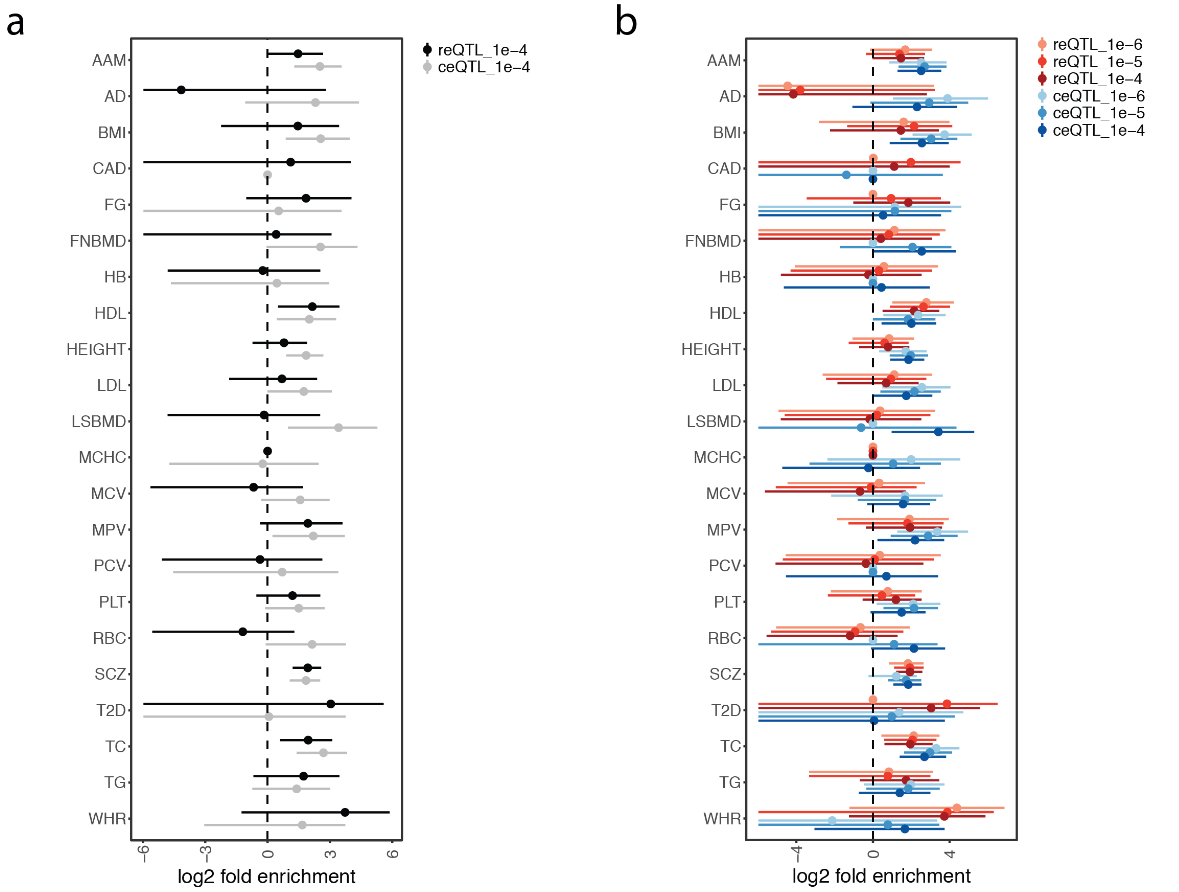

Supplementary Figure 11. Contribution of reQTLs to 22 non-autoimmune traits. **(a)** Fgwas analysis of reQTL and ceQTL associations in GWAS with 95% confidence intervals. x-axis is truncated at -6 for ease of display. Trait abbreviations: Age at menarche (AAM), Alzheimer's disease (AD), body mass index (BMI), coronary artery disease (CAD), fasting glucose (FG), bone mineral density (femoral neck) (FNBMD), hemoglobin (HB), high-density lipoproteins (HDL), height (HEIGHT), low-density proteins (LDL), bone mineral density (lumbar spine) (LSBMD), mean cell hemoglobin concentration (MCHC), mean red cell volume (MCV), mean platelet volume (MPV), packed red cell volume (PCV), platelet count (PLT), red blood cell count (RBC), schizophrenia (SCZ), type 2 diabetes (T2D), total cholesterol (TC), triglycerides (TG), waist-hip ratio (WHR). **(b)** Same fgwas analysis as in **(a)** using various eQTL p-value cutoffs yields similar estimates. Estimates where the confidence interval could not be calculated were set to 0. For some diseases where monocytes are unlikely to be the relevant cell type, the enrichment of ceQTLs may reflect eQTLs that are shared between diverse cell types and conditions.

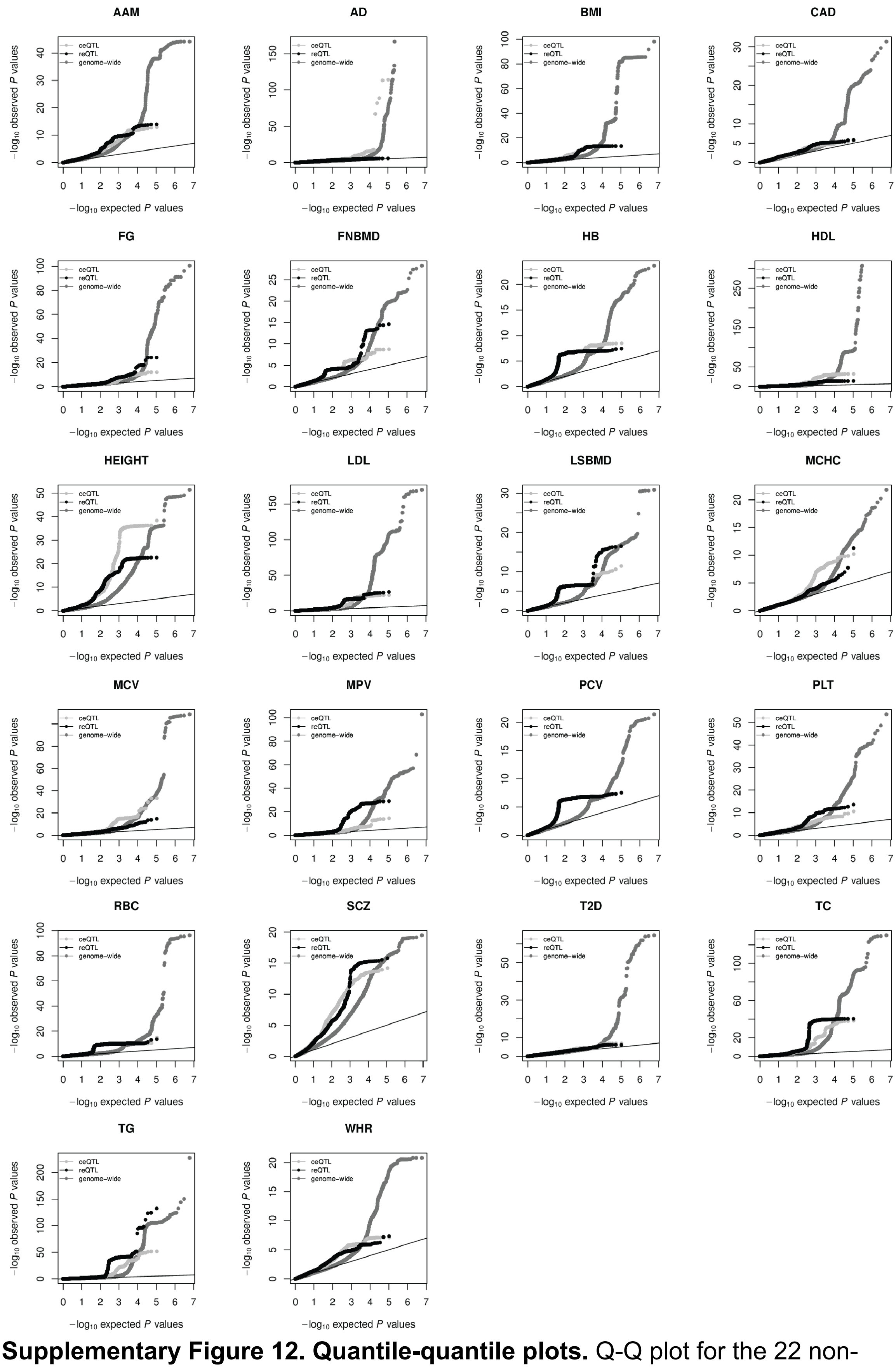

Supplementary Figure 12. Quantile-quantile plots. Q-Q plot for the 22 non-autoimmune traits tested for enrichment of reQTLs and ceQTLs in Supplementary Fig. 11. See figure legend of Supplementary Fig. 11 for trait abbreviations. All hits in the extended MHC region (26Mb–34Mb on hg19 chromosome 16) were removed.

Supplementary References

1. Fairfax, B. P. *et al.* Innate Immune Activity Conditions the Effect of Regulatory Variants upon Monocyte Gene Expression. *Science* **343,** 1246949–1246949 (2014).
2. Lee, M. N. *et al.* Common Genetic Variants Modulate Pathogen-Sensing Responses in Human Dendritic Cells. *Science* **343,** 1246980–1246980 (2014).
3. Herwig, R., Hardt, C., Lienhard, M. & Kamburov, A. Analyzing and interpreting genome data at the network level with ConsensusPathDB. *Nat Protoc* **11,** 1889–1907 (2016).
4. Szklarczyk, D. *et al.* STRING v10: protein-protein interaction networks, integrated over the tree of life. *Nucleic Acids Res* **43,** D447–D452 (2015).
